# Prandtl Number in Classical Hard-Sphere and One-Component Plasma Fluids

**DOI:** 10.3390/molecules26040821

**Published:** 2021-02-05

**Authors:** Sergey Khrapak, Alexey Khrapak

**Affiliations:** Joint Institute for High Temperatures, Russian Academy of Sciences, 125412 Moscow, Russia; khrapak@mail.ru

**Keywords:** transport properties of fluids, Prandtl number, hard-sphere system, one-component plasma, thermal conductivity of fluids

## Abstract

The Prandtl number is evaluated for the three-dimensional hard-sphere and one-component plasma fluids, from the dilute weakly coupled regime up to a dense strongly coupled regime near the fluid-solid phase transition. In both cases, numerical values of order unity are obtained. The Prandtl number increases on approaching the freezing point, where it reaches a quasi-universal value for simple dielectric fluids of about ≃1.7. Relations to two-dimensional fluids are briefly discussed.

The Prandtl number, Pr, is a dimensionless quantity characterizing the ratio of momentum-to-thermal diffusivity and is defined as [1,2]

(1) $$\mathrm{Pr}=\frac{c_{p}\eta }{m\lambda },$$

where η is the shear viscosity coefficient, λ is the thermal conductivity coefficient, *m* is the atomic mass, and $c_{p}=C_{p}/Nk_{B}$ is the reduced specific heat. The Prandtl number is a useful intrinsic property, which can vary considerably depending on a substance. For instance, for liquid metals Pr≪1 due to electron contribution to thermal conductivity. Usually, the Prandtl number is around 0.7 for gases, around 6.9 for water at 25 ${}^{\circ }$C, and can be higher for liquified noble gases [2]. Various oils are characterized by very high values of Prandtl number, Pr≫1. Additionally, the Prandtl number can depend on thermodynamic conditions.

The purpose of this Brief Report is to investigate how the softness of the interparticle interaction can affect the Prandtl number. We evaluate the magnitude of the Prandtl number for two model fluids in three dimensions with very different pairwise interaction potentials across their corresponding phase diagrams. The hard-sphere (HS) fluid represents the steepest pairwise interaction while the one-component plasma (OCP) model represents the opposite limiting case of very soft long-range interaction.

The HS model is widely used to approximate structural and transport properties of simple gases, fluids and solids [3]. The interaction potential between the two hard spheres of diameter σ, whose centers are separated by the distance *r*, is infinitely large at r<σ (impenetrable condition) and is zero otherwise. This mimics the strong repulsion that atoms experience at short separations. The OCP model is an idealized system of point charges immersed in a neutralizing uniform background of opposite charge (e.g., ions in the immobile background of electrons or vice versa) and interacting via a very soft Coulomb potential energy, $e^{2}/r$, where *e* is the electric charge [4,5,6]. This model is of considerable practical interest in the context of charged (ionized) matter, including laboratory and space plasmas, planetary interiors, white dwarfs, liquid metals, and electrolytes, as well as colloidal suspensions and complex (dusty) plasmas [7,8,9]. Both HS and OCP systems are important reference models to test and validate various theories and approximations developed for condensed phases. In particular, a proper comparison can help to understand the effects of potential softness on various processes and phenomena.

Structure and thermodynamics of HS and OCP systems are in each case characterized by a single dimensionless parameter. In the case of HS system this is the packing fraction, $\phi =\left(\pi /6\right)\rho \sigma^{3}$, where ρ is the particle density. The HS fluid behaves similar to an ideal gas at ϕ≪1 and exhibits the fluid-solid phase transition at higher density, with the freezing (melting) point at $\phi_{f}≃0.494$ ($\phi_{m}≃0.545$) [10]. In the OCP case, the coupling parameter $\Gamma =e^{2}/aT$, where $a={\left(4\pi \rho /3\right)}^{-1/3}$ is the Wigner-Seitz radius, and *T* is the temperature in energy units ($k_{B}=1$), is traditionally used to describe the phase state. The ideal-gas behavior corresponds to Γ≪1, while at Γ≳1 the OCP demonstrates liquid-like properties [11]. The fluid-solid phase transition occurs at Γ≃174 [6,12,13].

Transport properties of the HS and OCP fluids are very well investigated in classical MD simulations. Extensive data on the self-diffusion, shear viscosity, and thermal conductivity coefficients have been published and discussed in the literature. For the HS system, we use the recent comprehensive simulation study by Pieprzyk et al. [14,15]. For the OCP model we use the shear viscosity data tabulated by Daligault et al. [16] and thermal conductivity data tabulated by Scheiner and Baalrud [17]. When expressed using Rosenfelds’ reduced units [18,19], the shear viscosity $\eta_{R}=\eta \rho^{-2/3}/mv_{T}$ and the thermal conductivity $\lambda_{R}=\lambda \rho^{-2/3}/v_{T}$ coefficients have the following dependence on the HS packing fraction and the coupling parameter (here $v_{T}=\sqrt{T/m}$ is the thermal velocity). In the dilute gaseous (weakly coupled) limit they decrease with ϕ and Γ until the minimum is reached at ϕ∼0.1 and Γ∼10, respectively. Then $\eta_{R}$ and $\lambda_{R}$ increase with ϕ and Γ. The values of $\eta_{R}$ at the freezing point of HS and OCP fluids are comparable (∼5). The thermal conductivity coefficient $\lambda_{R}$ is somewhat higher for the HS fluid (∼15) than for the OCP fluid (∼10) at freezing, according to the simulation results.

In addition to viscosity and thermal conductivity data obtained previously in numerical simulations we need the specific heat values to calculate the Prandtl number (1). The following thermodynamic identities are used for this purpose [20]:

(2a) $$\begin{array}{c} C_{V}={\left(\frac{\partial U}{\partial T}\right)}_{V}, \end{array}$$

(2b) $$\begin{array}{c} C_{P}-C_{V}=-T\frac{{\left(\partial P/\partial T\right)}_{V}^{2}}{{\left(\partial P/\partial V\right)}_{T}}, \end{array}$$

where *U* is the internal energy, *P* is the pressure, and V=N/ρ is the system volume.

For HS fluids, the internal energy is only due to kinetic contribution, U/NT=3/2. This implies that the reduced specific heat is just constant, $c_{V}\equiv 3/2$. For the pressure we use the Carnahan-Starling (CS) equation of state (EoS) [21]:

(3) $$\frac{P}{\rho T}=\frac{1+\phi +\phi^{2}-\phi^{3}}{{\left(1-\phi \right)}^{3}}.$$

For the OCP we use a simple EoS proposed in Ref. [22], based on extensive Monte Carlo simulation data from Ref. [23]. The internal energy in this approximation reads

(4) $$\frac{U}{NT}≃\frac{3}{2}-\frac{9}{10}\Gamma +0.5944\Gamma^{1/3}-0.2786.$$

The first term on the right-hand-side is the kinetic energy, the second term corresponds to the so-called ion sphere model (ISM) [6,12,24], which dominates at strong coupling. ISM assumes that due to the strong Coulomb repulsion between the particles they form a regular quasi-crystalline structure. The system can then be approximated as a collection of particles, each attached to a spherical piece of the uniform background of radius *a*, which exactly compensates the particle charge. These compound charge-neutral spheres are not overlapping and the system energy is just the sum of the energies of the individual spheres. The latter can be easily calculated via purely electrostatic arguments. The last two correction terms in (4) improve the agreement with the numerical results [22,25]. The pressure corresponding to EoS of Equation (4) is

(5) $$\frac{P}{\rho T}≃1-\frac{3}{10}\Gamma +0.1981\Gamma^{1/3}-0.0929,$$

where we have used the fact that the excess (over the ideal gas) components of the pressure and energy are related via $p_{\mathrm{ex}}=\frac{1}{3}u_{\mathrm{ex}}$.

The subsequent calculation is straightforward and the results are presented in Figure 1 and Figure 2.

In Figure 1, the packing fraction of the HS fluid varies from ϕ≃0.05 to $≃\phi_{f}$. We observe a minimum Pr≃0.6 at ϕ≃0.1. In the dilute limit we have $c_{p}=5/2$, $\eta =\left(5/16\sigma^{2}\right){\left(mT/\pi \right)}^{1/2}$, and $\lambda =\left(75/64\sigma^{2}\right){\left(T/\pi m\right)}^{1/2}$, so that Pr=2/3 (this results is independent on the interatomic interaction potential) [1]. This asymptote is approached from below. For ϕ≳0.1 the Prandtl number increases monotonically and reaches Pr≃2 at freezing.

Similarly, in the strongly coupled OCP (Γ≫1), the Prandtl number increases monotonically and reaches Pr≃1.5 at freezing. The range of Γ shown in Figure 2 starts from Γ=10, to avoid the region around Γ≃3, where the isothermal compressibility changes sign, and hence $c_{p}$ and $c_{p}/c_{v}$ diverge. At very weak coupling, Γ≪1, the Prandtl number should approach the ideal gas asymptote 2/3.

Thus, in both HS and OCP fluids, the Prandtl number is around unity, indicating that momentum and thermal diffusivities are comparable in magnitude. Close values of the Prandtl number at the freezing of two fluids with quite different potentials of interaction points to a possible quasi-universality involved. The following consideration supports this conjecture. The Andrade’s scaling [26,27] for the liquid shear viscosity at the melting point can be cast in the form

(6) $$\eta_{m}=C\rho^{2/3}{\left(T_{m}m\right)}^{1/2},$$

where *C* is a number that varies between 5 and 6 for various liquid metals; C≃4.8 for a soft repulsive Yukawa system; C≃5.2 for a Lennard-Jones system, and C≃5.8 for a liquefied argon at the melting temperature [28]. We take an “average” value C=5.5 for further estimates. A vibrational model of heat conduction, discussed recently [29], relates the heat conductivity coefficient to specific heat, density, and sound velocities of a fluid:

(7) $$\lambda ≃0.16c_{v}\rho^{2/3}\left(c_{l}+2c_{t}\right),$$

where $c_{l}$ and $c_{t}$ are the longitudinal and transverse sound velocities. For a rough simple estimate we assume $c_{l}∼2c_{t}$, which is approximately so for inverse power law interactions with sufficiently large exponents, Lennard–Jones fluids, and some liquid metals [30]. Combining Equations (6) and (7) we get

(8) $$\mathrm{Pr}≃17.2\frac{c_{p}}{c_{v}}\frac{v_{T}}{c_{l}}{|}_{T=T_{m}}.$$

The last step is to neglect the difference between $c_{p}$ and $c_{v}$ at melting conditions (which is particularly appropriate for soft repulsive interactions and less appropriate for HS interactions) and use the fact that ${\left(c_{l}/v_{T}\right)}_{T_{m}}≃10$ for many simple melts, including Lennard–Jones, soft-spheres, hard-spheres, and liquid metals [31,32,33]. We obtain Pr≃1.7 at the melting temperature. Note that although the arguments presented above apply directly neither to the HS system (due to strong anharmonicity) nor to the OCP system (due to non-acoustic character of the longitudinal dispersion relation), they should be relevant to a wide class of intermediate pairwise interactions in simple dielectric fluids. Remarkably, these arguments yield the Prandtl number at freezing just in between the values obtained for the HS and OCP limiting regimes.

Our current understanding of transport phenomena in two-dimensional (2D) systems is not sufficient to perform a detailed comparison similar to that in three dimensional (3D) systems, presented above. The problem remains rather controversial. The absence of valid transport coefficients in 2D systems was predicted based on the analysis of the velocity autocorrelation function and of the kinetic parts of the correlation functions for the shear viscosity and the heat conductivity [34]. However, in some special cases finite transport coefficients have been reported in simulations and experiments. This is for instance the case of 2D Yukawa systems, which represent important model systems with relations to aqueous solutions of electrolytes, charged colloidal micro- and nano-particles in various solvents and at interfaces of fluid media, as well as charged particles in complex plasmas [7,8,35]. The Yukawa potential—Coulomb potential with exponential screening—represents the case intermediate between the Coulomb potential and the hard-sphere potential, which is approached at (unrealistically) strong screening. Existence of finite shear viscosity coefficients of strongly coupled 2D Yukawa fluids was reported in experiments with complex (dusty) plasma monolayers and molecular dynamics (MD) simulations [36,37,38,39,40]. Finite values of the thermal conductivity coefficient were reported for single layers of complex plasma in experiments [41,42,43] and numerical simulations [44,45,46]. A critical analysis of these data along with a comparison with the vibrational model of thermal conduction adapted to the 2D geometry has been performed [47].

Our present knowledge allows us to make only a rough estimate. According to the data shown in Figure 3 of Ref. [38], the shear viscosity coefficient near the freezing transition can be estimated as $\eta ≃m\rho \Omega_{E}a^{2}$, where $\Omega_{E}$ is the characteristic Einstein frequency (and $a=1/\sqrt{\pi \rho }$ is a 2D Wigner-Seitz radius). On the other hand, the thermal conductivity coefficient tends to $\lambda ≃\rho \Omega_{E}a^{2}$ [47] as freezing is approached. The 2D analog of the Dulong-Petit law implies $c_{v}≃2$ in 2D solids and dense fluids. The difference between $c_{v}$ and $c_{p}$ can be neglected at strong coupling (see Figure 3 from Ref. [48]). As a result, we obtain that Pr≃2 near the fluid–solid phase transition of 2D Yukawa fluids, comparable to the numerical values of 3D fluids.

Experimental measurement of the Prandtl number (along with Brinkman and Eckert numbers) was performed in a complex plasma subject to a laser-driven flow [49]. The reported value of Pr≃0.09 appears too small, compared to what one would expect on the basis of theoretical consideration above. We note, however, that in this experiment profound peaks of the particles’ kinetic temperature were observed. These occurred in the regions of high-velocity shear and were attributed to viscous heating. Particle heating resulted in weaker coupling and this could lower the Prandtl number compared to its freezing point value. Additionally, the shear viscosity coefficient we have used corresponds to the simulations with small shear rates, which agree with equilibrium MD simulations [38]. The high shear rate simulations demonstrated that the shear viscosity coefficient can be significantly reduced for these conditions in a manifestation of shear thinning [38,39]. This might be the reason for the observed discrepancy.

The main results can be summarized as follows: (i) the steepness of the pairwise interatomic interaction in simple fluids does not affect considerably the magnitude of the Prandtl number; (ii) for simple pairwise repulsive interactions in 3D the Prandtl number is about unity and increases in the dense fluid regime; (iii) it reaches a quasi-universal value of ≃1.7 at the freezing of simple dielectric fluids; (iv) comparable values are expected for 2D fluids, the experimental value available for a complex plasma is however lower, probably due to shear thinning effect.

## Figures and Tables

**Figure 1:**
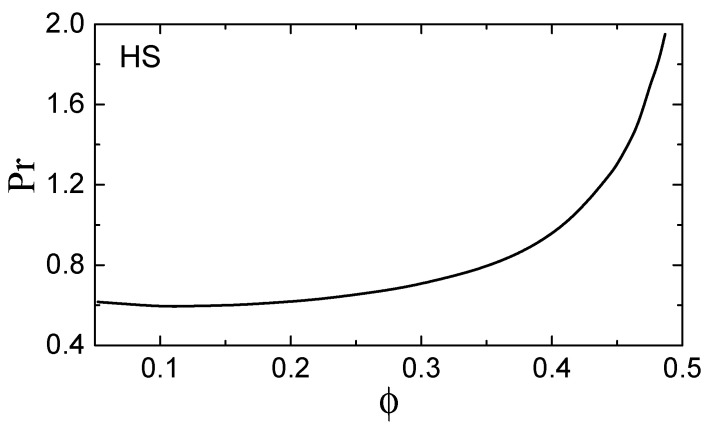
Prandtl number, Pr, of a hard-sphere (HS) fluid versus the packing fraction ϕ.

**Figure 2:**
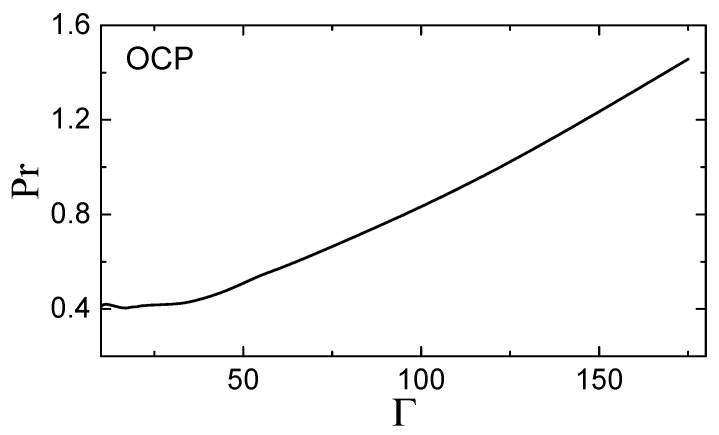
Prandtl number, Pr, of a strongly coupled one-component plasma (OCP) fluid versus the coupling parameter Γ.

## Data Availability

The data that support the findings of this study are available from the corresponding author, S.K., upon reasonable request.
